# CircCOPS8 Inhibits the Proliferation of Buffalo Myoblasts by Binding to IGF2BP3 and Promoting ATR Gene Expression

**DOI:** 10.3390/ani16071017

**Published:** 2026-03-26

**Authors:** Yuting Dou, Ping Sun, Xiangping Cheng, Mengjie Chen, Xinxin Li, Jieping Huang, Zhipeng Li, Qingyou Liu, Deshun Shi, Hui Li, Jian Wang

**Affiliations:** 1 Guangxi Key Laboratory of Animal Breeding, Disease Control and Prevention, College of Animal Science and Technology, Guangxi University, Nanning 530004, China; douyuting9811@163.com (Y.D.); 14795746742@163.com (P.S.);; 2 Hubei Key Laboratory of Animal Embryo Engineering and Molecular Breeding, Institute of Animal Husbandry and Veterinary, Hubei Academy of Agricultural Sciences, Wuhan 430064, China; 3 Institute of Scientific Research, Guangxi University, Nanning 530004, China; 4 College of Animal Science and Technology, South China Agricultural University, Guangzhou 510640, China

**Keywords:** muscle proliferation, CircRNAs, CircCOPS8, IGF2BP3

## Abstract

Buffaloes are vital livestock in South-East Asia, attributed to their adaptation to hot and humid climates as well as their capacity to produce high-quality milk and meat. However, the texture of buffalo meat is suboptimal and its slow growth rate restricts the development of the buffalo farming industry. Consequently, studies exploring the key biochemical factors associated with buffalo muscle development have become a research focus. CircRNAs are a class of non-coding RNAs which can function as molecular sponges, participate in protein scaffold formation, and encode short peptides. Previous studies have shown that circRNAs are capable of regulating muscle development; however, relatively few reports have addressed their association with buffalo muscle development. In this study, data from Western blotting and RT-qPCR showed that circCOPS8 significantly enhanced the differentiation of buffalo myoblasts while inhibiting their proliferation (*p* < 0.05). In contrast, in a mouse model of muscular injury, circCOPS8 prevented the repair of injured muscles. Additionally, RIP-qPCR assays confirmed that circCOPS8 could bind to IGF2BP3 (*p* < 0.05). Furthermore RT-qPCR and transcriptome sequencing results revealed that circCOPS8 inhibited cell growth by upregulating the expression of genes such as ATR (*p* < 0.05). Our findings suggested that circCOPS8 promoted the differentiation and apoptosis of buffalo myoblasts while inhibiting their proliferation. The inhibition of cell proliferation was primarily mediated by the binding of circCOPS8 to IGF2BP3 and the promotion of ATR gene expression. This study investigated the role and underlying mechanism of circCOPS8 in buffalo myoblasts, which will extend our understanding of non-coding RNA-mediated regulation of buffalo muscle development, with the ultimate goal of improving the meat quality of buffaloes.

## Simple Summary

Buffaloes play a crucial role in South-East Asia due to their ability to thrive in hot and humid climates while producing high-quality milk and meat. However, the slow growth and tough texture of buffalo meat pose significant challenges that impede the expansion of buffalo farming. To address these issues, researchers are studying the factors involved in buffalo muscle development, among which circular RNAs (circRNAs)—a type of RNA molecules capable of regulating muscle growth—are identified as a key factor. This study focuses on circCOPS8, a specific circRNA, and explores its regulatory role in buffalo muscle cells. The researchers found that circCOPS8 promotes the differentiation of buffalo muscle cells while inhibiting their growth. However, in a mouse model of muscle injury, circCOPS8 was shown to retard the process of muscle repair. Additionally, the study revealed that circCOPS8 interacts with the insulin-like growth factor 2 mRNA-binding protein 3 (IGF2BP3) and modulates the expression of genes associated with cell growth. The findings indicate that circCOPS8 facilitates the maturation of buffalo muscle cells and restricts their excessive growth. This research enhances our understanding of the regulatory mechanisms through which non-coding RNA affects buffalo muscle development, thereby providing potential strategies for improving the quality of buffalo meat.

## 1. Introduction

Swamp buffalo is a characteristic species of South-East Asia, where it thrives in a hot and humid climate, providing labor, milk and meat, thereby making it a vital livestock for the local communities [1,2]. Its large population makes the swamp buffalo a promising potential meat source, which is comparable to beef cattle with respect to its meat composition and organo-leptic characteristics [3]. In addition, buffalo meat is nutrient-dense, with high levels of polyunsaturated fatty acids and conjugated linoleic acid [4], and it is noted for its potential health benefits, such as its ability to reduce cardiovascular risk factors [5]. However, compared with cattle, the rib-eye area of buffalo carcasses is smaller, and its meat has less marbling, resulting in its inferior taste to the human palate. This has hindered the development of its farming potential and meat consumption [6].

Skeletal muscle makes up approximately 30 to 40% of the adult animal’s body weight and is an important component of the animal body [7]. During the embryonic stage, mesodermal cells will give rise to satellite cells of the skeletal muscles, which can multiply [8,9]. Skeletal muscle cells cease proliferate when they fuse with muscle fibers and myotubes [8,10,11]. The Pax3 gene activates cell growth in skeletal muscles during the embryonic stage [10]. Muscle cells in mammals that express Pax7 stop going through the cell cycle and have reduced Pax3 gene expression. Pax7 causes myoblast development by stimulating the expression of MyoD and Myf5 [12]. After differentiation, the cell membranes of myocytes dissolve and fuse with each other to form multinucleated myotubes [7,13]. To form mature muscle fibers, which eventually mature into contractile muscles, myotubes produce contractile proteins, such as actin and myosin [14]. Exploring the expression of key genes in adult muscle cells may contribute to improving meat production in farmed animals.

In recent years, emerging evidence indicated that circRNAs play an important regulatory role in skeletal muscle generation and development [15,16]. CircRNAs are a class of non-coding RNA molecules with closed-loop structures that lack 5′cap and 3′poly (A) structures, features that render them more stable and not easily degraded [17,18]. It has been shown that circRNAs can regulate the development of muscles by adsorbing miRNAs as molecular sponges, serving as protein scaffolds, and competitively interacting with certain proteins [19,20]. For instance, circNfix acted as a sponge for miR-204 to enhance MEF2C expression and promote myotube formation [21]. CircFAM188B-103AA, a peptide encoded by circFAM188B, stimulated the proliferation of chicken skeletal muscle satellite cells while inhibiting their differentiation [22]. CircHipk2 can bind to the ribosomal protein Rpl7 and inhibit myoblastic proliferation while promoting differentiation [23]. Although the role of circRNA in muscle development has been widely recognized, functional studies in the specific species of buffalo remain largely unexplored, and the functional mechanisms of many known circRNAs are still awaiting clarification.

Buffalo meat is firm and fibrous, and there is still a disparity in texture compared to beef [24]. Our previous RNA-seq findings (data number SAMN21421329) showed that the expression levels of circCOPS8 in the longissimus dorsi muscles of buffaloes were substantially higher than the levels in cattle. The COPS8 gene itself is a key component of the COP9 signalosome, a complex involved in ubiquitination processes and closely associated with cell cycle regulation, apoptosis, and other functions. Therefore, its derived circRNA likely plays a significant role. In this study, we considered whether this differentially expressed circRNA was likely to have some effect on the quality of buffalo meat. Therefore, we aimed to investigate the effects of circCOPS8 on buffalo myoblast proliferation, differentiation, apoptosis and muscle damage repair. We also sought to identify the regulatory pathway of circCOPS8 and analyze the potential molecular mechanism.

## 2. Materials and Methods

Ethical Statement: All animal experimental designs and procedures were in accordance with institutional guidelines and were approved by the Animal Ethics Committee of Guangxi University (approval number: GXU-2022-309).

### 2.1. Synthesis of cDNA and Real-Time qPCR

The longissimus dorsi muscles of 90-day-old Guangxi native buffalo fetuses were collected for cDNA preparation. TRIzol reagent was added to the tissues to extract total RNA (Life, San Fernando Valley, CA, USA). Then, 1–3 μL of 20 U/μL RNase R (Geneseed, Guangzhou, China) was added to 1 μg of total RNA and the mixture was incubated at 37 °C for 60 min. The RNA was converted into cDNA by using a HiScript III reverse transcription kit (Vazyme, Nanjing, China). Real-time PCR (Vazyme, Nanjing, China) was performed using AceQ qPCR SYBR Master Mix on an ABI 7500 machine (Thermo, Waltham, MA, USA). The primers used are shown in Table S1.

### 2.2. Construction Vector

The vector of plasmid pCD2.1-circCOPS8 was constructed. The cloned circCOPS8 sequence was ligated into a pCD2.1-ciR vector (Geneseed, Guangzhou, China) by using Kpn I and Bam HI restriction sites. The primers used are shown in Table S2.

### 2.3. Cell Isolation and Culture

The longissimus dorsi muscles of 90-day-old native Guangxi buffalo fetuses were washed with ultrapure water, PBS (containing 2% penicillin/streptomycin, Gibco, Merck Millipore, Burlington, MA, USA), 75% alcohol and PBS (containing 1% penicillin/streptomycin, Gibco, Merck Millipore, MA, USA) in turn. The muscles were cut into small pieces and then they were transferred into a 15 mL centrifuge tube. The tissue suspension was left to stand and the supernatant was discarded. Two volumes of collagenase I (Sigma, St. Louis, MO, USA) were added to the tissues and they were shaken at 2000 rpm for 1 h at 37 °C. The mixture was centrifuged at 1500 rpm for 5 min and the supernatants were discarded. The mixture was shaken at 37 °C for 20 min with 0.25% trypsin. Complete medium (Gibco, Merck Millipore, MA, USA) was added to stop digestion. The cells were filtered twice through 100 μm cell filters and then they were separated by centrifugation at 1500 rpm for 6 min. DMEM containing 20% fetal bovine serum (Gibco, Merck Millipore, MA, USA) and 1% penicillin/streptomycin (Gibco, Merck Millipore, MA, USA) was then added. After culturing in a cell incubator for 2 h (37 °C, 5% CO_2_), the dispersed cells were transferred into a new culture dish. This step was repeated twice. When the myoblast fusion had reached 80%, DMEM (Gibco, Merck Millipore, MA, USA) containing 2% horse serum was added in order to induce cell differentiation.

### 2.4. Fluorescence in Situ Hybridization (FISH)

FISH experiments were performed with the in situ hybridization reagents (Servicebio, Wuhan, China) according to the manufacturer’s instructions. Cell-climbing slices of buffalo myoblasts were constructed and the cells were cultured at room temperature for 30 min with FISH fixative. The cells were solubilized with 0.2% Triton X-100 PBS for 5 min at room temperature and then they were incubated with pre-hybridization solution for 1 h at 37 °C. The fixed cells were added to the fluorescently labeled hybridization solution of the circCOPS8 probe and then they were incubated in the dark at 4 °C overnight. After 24 h, the cells were washed 3–5 times with PBS, DAPI solution was added, and the cells were kept at room temperature for 5 min in a dark and humid atmosphere. The cells were observed and photographed using a fluorescence microscope (Nikon, Tokyo, Japan).

### 2.5. Cell Counting and Proliferation Assays

CCK8 and EdU assays were carried out using kits by following the manufacturer’s instructions. The cells were treated according to the instructions for the CCK-8 kit (Vazyme, Nanjing, China), and the absorbance was measured at 450 nm by using a microplate reader. The Apollo 567 (Ribobio, Guangzhou, China) was used to measure the results from the EdU assays. This measured the proliferation of buffalo skeletal muscle cells, and the cells were observed and photographed by using a fluorescence microscope (Nikon, Tokyo, Japan). The software package used for cell cycle analysis was ModFit LT 5.0.

### 2.6. Western Blotting

Herein, 1% PMSF RIPA lysate buffer (Solarbio, Beijing, China) was added to cell and tissues to extract the proteins. The BCA kit (Solarbio, Beijing, China) was used to determine the protein concentration. Proteins were separated by 10% SDS-PAGE (Bio-RAD, Hercules, CA, USA) electrophoresis and they were then transferred to nitrocellulose membranes (PALL, New York, NY, USA). The membranes were incubated with primary antibodies (ABclonal, Wuhan, China) specific for cyclinD1 (Catalog: A11310), PCNA (Catalog: A0264), MyoG (Catalog: A11368), MyoD (Catalog: A23881) and β-actin (Catalog: AC038) overnight at 4 °C. The recommended dilutions used for cyclinD1, PCNA, MyoG, MyoD and β-actin were 1:500–1:1000, 1:500–1:1000, 1:500–1:2000, 1:500–1:1000 and 1:10,000–1:100,000, respectively. The secondary antibody used was goat-anti-rabbit IgG (ABclonal, Wuhan, China, Catalog: AS014), and the dilution ratio was 1:2000–1:10,000. After the first incubation, the membranes were washed with TBS and TBST, and the secondary antibody (ABclonal, Wuhan, China) was incubated for 1 h at room temperature. After washing, ECL Plus chromogenic solution (Solarbio, Beijing, China) was used and the image obtained was captured by using the ChemiDoc XRS+ system (Bio-Rad, Hercules, CA, USA).

### 2.7. Establishment of Skeletal Muscle Injury Model

Although we compared the sequences of circCOPS8 in mice and buffaloes, the generation mechanism for circCOPS8 production is still unclear. The experimental mice used were 4–5-week-old SPF-grade Kunming mice (n = 12) purchased from the Experimental Center of Guangxi Medical University. The anterior tibia muscle of mice was injected with 50 μL myocardial toxin solution (CTX) 48 h later and the tissues were frozen. Sections were cut and H&E stain was used to assess the muscle injury. After the creation of effective muscle damage models, the mice were injected with the pCD2.1-circCOPS8 plasmid. The in vivo transfection reagent complex (Engreen Bio-system Co., Ltd., Beijing, China) was injected into the left anterior tibia muscle and this was the experimental group. A combination of the in vivo transfection reagent and pCD2.1 plasmid was injected into the mouse right anterior tibia muscle, and this served as the control group. Three injections were administered at 12, 48 and 96 h after the CTX-induced muscle injury. After 144 h of CTX treatment, RNA was extracted from samples of the tibia muscles and reverse-transcribed. The Animal Care Committee of the College of Animal Science and Technology of Guangxi University (GXU-2022-309) approved the research protocol and animal care procedures. The ethical approval license (IACUC) granted was 202209220.

### 2.8. H&E Staining and Tissue Immunofluorescence

The collected tibialis anterior samples were subjected to H&E staining and tissue immunofluorescence. The tissues were fixed with a 4% neutral formaldehyde fixative for 24 h and subjected to gradient dehydration with ethanol. The tissues were trimmed into 0.5 cm^3^ cubes and immersed in a 1:1 mixture of OCT embedding medium (SAKURA, Osaka, Japan) and 30% sucrose at room temperature for 2 h. The tissues were then transferred to new OCT embedding medium and they were immersed at room temperature for 6 h. The tissues, together with the OCT embedding medium, were frozen and sectioned with a microtome to a thickness of 510 μm.

The sections were incubated with 0.5% Triton X at room temperature for 20 min and blocked with 10% BSA at room temperature for 1 h. They were then incubated overnight at 4 °C after the addition of primary antibodies, cyclinD1, PCNA, MyoD and MyHc (ABclonal, Wuhan, China). The tissue sections were washed with PBS three times and then they were incubated with secondary antibodies at room temperature for 60 min. After a 15 min incubation in 0.1% DAPI solution, they were washed 3 times with PBS. Finally, the fluorescence signals were observed under a fluorescence microscope (Leica, Wetzlar, Germany). The average fluorescence intensity of the protein (n = 7) was analyzed and counted using Image J 2 software. The data were then expressed as means ± SEMs.

### 2.9. Website Prediction

The miRNA adsorption capacity was predicted by the miRNA database. OFR Finder and the IRESite and SRAMP websites were used to predict the translation ability of circCOPS8. The amino acid sequence encoded by circCOPS8 was compared with the protein database, NMDC. RBPsuite and catRAPID were used to predict the proteins that circCOPS8 might bind to. The upstream pathway genes of proliferation marker genes were found through the KEGG website. The URLs of these websites are given in Supplementary Figure S3.

### 2.10. RIP-qPCR

Buffalo myoblasts that had been transfected with pCD2.1-circCOPS8 and pCD2.1-ciR plasmids were collected by following the kit manufacturer’s instructions (Geneseed, Guangzhou, China). IGF2BP3 (ABclonal, Wuhan, China, Catalog: A4444) was predicted by the website and it was selected as the protein that circCOPS8 might bind to. The dilution ratio of rabbit control IgG (ABclonal, Wuhan, China, Catalog: AC005) used was 1:50–1:200. The eluted and purified RNA was analyzed by qPCR. The primers used are given in Supplementary Table S1.

### 2.11. Preparation of Sequencing Samples

RNA was extracted from tissues and cells from the two groups of buffalo myoblast samples, including the experimental group transfected with the pCD2.1-circCOPS8 plasmid and the control group transfected with the pCD2.1-ciR plasmid. There were three biological replicates in each group. The completeness and total amount of extracted RNA were measured by using the Agilent 2100 bioanalyzer (Agilent, Santa Clara, CA, USA).

### 2.12. Library Construction and Quality Inspection

The database was constructed using the total RNA as the initial RNA. After mRNA with a poly A tail was enriched with Oligo(dT) magnetic beads, a database was constructed according to the general and chain-specific database construction methods. After the library construction was completed, the library was diluted to 1.5 ng/μL using a Qubit 2.0 Fluorometer for preliminary quantification (Thermo, Waltham, MA, USA). Then the insert size of the library was assessed by using the Agilent 2100 Bio-analyzer (Agilent, Santa Clara, CA, USA). After the insert size met the required expectation, the effective concentration of the library was quantified by qRT-PCR. The effective concentration to ensure the quality of the library was higher than 2 nM.

### 2.13. Sequencing

After library quantification, Illumina (San Diego, CA, USA) was used to sequence different libraries according to the requirements of the effective concentration and target out-of-machine data volume. Four fluorescently labeled dNTP, DNA polymerase and adapter primers were added to the sequencing flow cell for amplification of the samples. When each sequencing cluster extended the complementary strand, the corresponding fluorescence could be released by adding a fluorescently labeled dNTP. The sequencer was used to obtain the information for the measured segments by capturing the fluorescence signals and converting the optical signals obtained to the sequenced peaks by using the computer software.

### 2.14. Data Quality Control

The sequenced fragments were converted into sequence data (as reads) by base recognition of CASAVA from the image data obtained by the high-throughput sequencing instrument. The raw data obtained by sequencing contained a small number of reads with sequencing linkers or low sequencing quality. To ensure the quality and reliability of the data analysis, the raw data needed to be filtered. This mainly included the removal of reads with adapters, those reads containing N (where N indicated that the base information could be determined) and the ones with low-quality reads (reads in which the number of bases of Qphred ≤ 20 accounted for more than 50% of the entire read length). The Q20, Q30 and GC contents of the clean data were then calculated. All subsequent analyses were based on the high-quality analysis performed by the clean data.

### 2.15. Sequence Assembly and Expression Evaluation of Transcripts

The reference genome and gene model annotations were downloaded. The reference genome was indexed using HISAT2 v2.0.5 and paired terminal clean reads were compared to the reference genome using HISAT2 v2.0.5. The FPKM was calculated for each gene using feature counts.

### 2.16. Identification of Differential Genes and Functional Annotations

The difference in expression between the two comparative combinations was analyzed using DESeq2 software (v 1.20.0). The resulting *p*-values were adjusted using the method of Benjamini and Hochberg to control the false detection rate. Genes with *p* values < 0.05 were marked as significantly different by using the DESeq2 software package. The *p*-values were adjusted by using the Benjamini and Hochberg method. The corrected *p*-values and |log2foldchange| were used as thresholds for the corrected differential expression.

### 2.17. Differential Gene Enrichment Analysis

GO and KEGG enrichment analysis of differentially expressed genes were implemented by the clusterProfiler R package (v 3.4.4).

### 2.18. Statistical Analysis

The difference between the two groups was statistically significant using the multi-*t* test (FDR < 0.01) of Prism v 6.01 software. *p* < 0.05 was considered significant and *p* < 0.01 was extremely significant.

## 3. Results

### 3.1. Authenticity Verification and Vector Construction of circCOPS8

**Figure 1:**
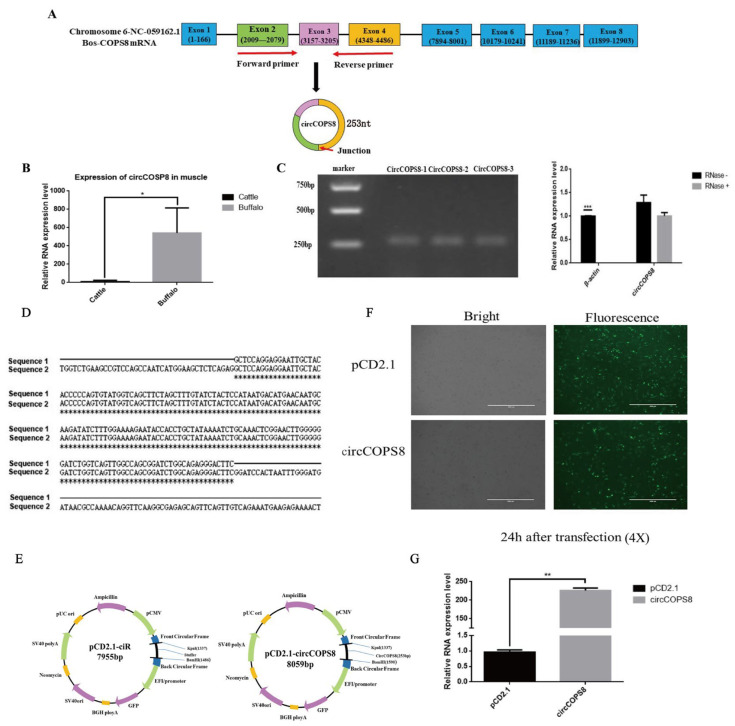
Composition and vector construction of circCOPS8. (**A**) CircCOPS8 is 253 nt in size and derived from exons 2, 3 and 4 of the gene COPS8. (**B**) The expression of circCOPS8 in the longissimus dorsi muscle of buffalo and cattle. * *p* < 0.05. (**C**) Nucleic acid gel electrophoresis of PCR products of circCOPS8 using RNase-digested cDNA from buffalo longissimus dorsi muscle as a template. *** *p* < 0.001. (**D**) The circCOPS8 sequence alignment map (Asterisks: The bases are completely conserved in the sequence alignment). (**E**) A vector overexpressing the plasmid pCD2.1-circCOPS8 was constructed. (**F**) Green fluorescence in transfected aqueous buffalo myoblasts with the plasmids pCD2.1-ciR and pCD2.1-circCOPS8 (scale bar: 1000 μm). (**G**) The overexpression efficiency of circCOPS8 was validated via qPCR. ** *p* < 0.01.

CircCOPS8 is derived from buffalo COPS8 (also known as COP9) which is localized on chromosome 18 and formed by splicing together the transcripts of exons 2, 3 and 4 of COPS8. It is composed of 253 nt (Figure 1A). Earlier RNA-seq investigations showed that the expression levels of circCOPS8 in buffaloes was significantly higher than those in cattle (Figure 1B). The total RNA was extracted from buffalo muscle cells and subjected to digestion with RNase R to verify the authenticity of circCOPS8. Since cicrRNAs are less prone to digestion than linear RNAs, agarose gel electrophoresis was used to verify the authenticity of circCOPS8. Subsequent RT-qPCR results showed that the expression levels of actin were significantly reduced after RNase R digestion, while those of circCOPS8 remained unchanged (Figure 1C). The amplified circCOPS8 was found to be compatible with the sequenced results, based on the findings of the plasmid sequencing verification (Figure 1D). Subsequently, we constructed a circCOPS8 vector and transfected it into buffalo myoblasts. RT-qPCR results confirmed that circCOPS8 was successfully overexpressed in buffalo myoblasts (Figure 1E–G).

### 3.2. CircCOPS8 Inhibits Myoblast Proliferation

**Figure 2:**
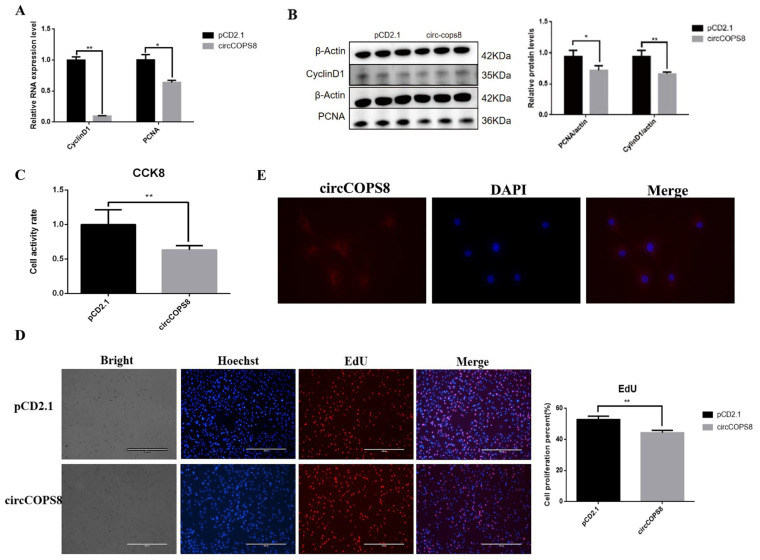
CircCOPS8 inhibits the proliferation of buffalo myoblasts. (**A**) Detection of expression levels of cell proliferation-related marker genes. (**B**) Western blotting to detect the expression levels of the proliferation marker gene and analysis of the gray-scale statistics. (**C**) CCK-8 cell proliferation experiment assays (n = 5). (**D**) EdU detects the proliferation of buffalo myoblasts. Blue represents the nucleus of all cells, and red represents the nucleus containing EdU. * *p* < 0.05, ** *p* < 0.01. (scale bar: 400 μm). (**E**) FISH detects the position of circCOPS8 in buffalo myoblasts, with red representing circCOPS8 and blue representing the nucleus.

**Figure 3:**
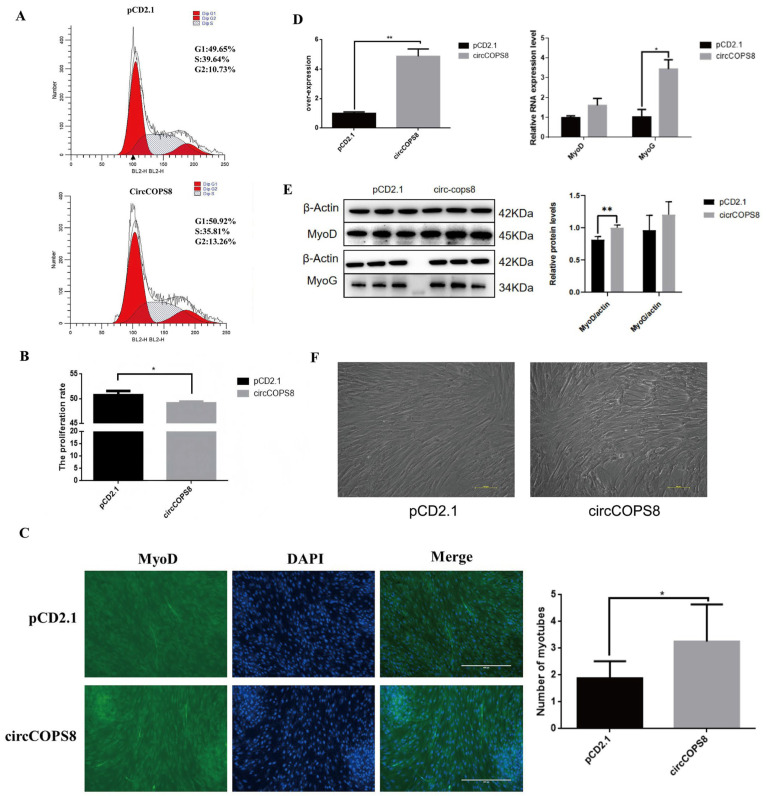
CircCOPS8 promotes the differentiation of buffalo myoblasts. (**A**) Cell cycle profile by flow cytometry. (**B**) The proliferation of buffalo myoblasts. (**C**) The effect of circCOPS8 on the differentiation of buffalo myoblasts was detected by cellular immunofluorescence (n = 8) (scale bar: 400 μm). (**D**) The overexpression efficiency of circCOPS8 in buffalo myoblasts and the expression level of differentiation marker genes were detected. (**E**) Western blotting to detect the expression levels of the proliferation marker gene and analysis of the gray-scale statistics. ** *p* < 0.01. (**F**) A myotube diagram of buffalo myoblast differentiation induced by overexpression of circCOPS8. * *p* < 0.05 (scale bar: 400 μm).

To investigate the effects of circCOPS8 on buffalo myoblast proliferation, RT-qPCR and Western blotting were performed to assess the mRNA and protein expression levels of cyclinD1 and PCNA, respectively. CyclinD1 marks the initiation of proliferation, while PCNA indicates the execution of proliferation. Together, they comprehensively reflect the dynamic process of cell proliferation. CyclinD1 and PCNA mRNA were significantly decreased by approximately 90 and 36%, and the protein expression was decreased by approximately 25 and 20%, respectively, while circCOPS8 was overexpressed (Figure 2A,B). However, the proliferation rate of buffalo myoblasts dropped dramatically as evidenced by the results of EdU and CCK-8 assays (Figure 2C,D). FISH analysis revealed that circCOPS8 is localized in the cytoplasm of buffalo myoblasts (Figure 2E). Based on the results of cell cycle detection by flow cytometry, overexpression of circCOPS8 reduced the number of cells in the G2 and S phases and suppressed cell proliferation (Figure 3A,B). In summary, circCOPS8 reduced the proliferation of buffalo myoblasts significantly.

### 3.3. CircCOPS8 Promotes the Differentiation of Myoblasts

To investigate the effects of circCOPS8 on buffalo myoblast differentiation, RT-qPCR and Western blotting were performed to assess the mRNA and protein expression levels of MyoD and MyoG, respectively. After effectively overexpressing circCOPS8 in buffalo myoblasts, differentiation of the myoblasts was induced (Figure 3B,F). The mRNA and protein expression of cell differentiation marker genes, including MyoD and MyoG, were increased. Specifically, MyoG mRNA expression was increased by approximately 244% and MyoD protein expression was increased by approximately 20% (Figure 3D,E). In accordance with the results obtained from cell immunofluorescence, the number of myotubes in the experimental group was higher than in the control group (Figure 3C). Therefore, circCOPS8 promoted buffalo myoblast differentiation.

### 3.4. CircCOPS8 Inhibits the Repair of Mouse Muscle Cells

**Figure 4:**
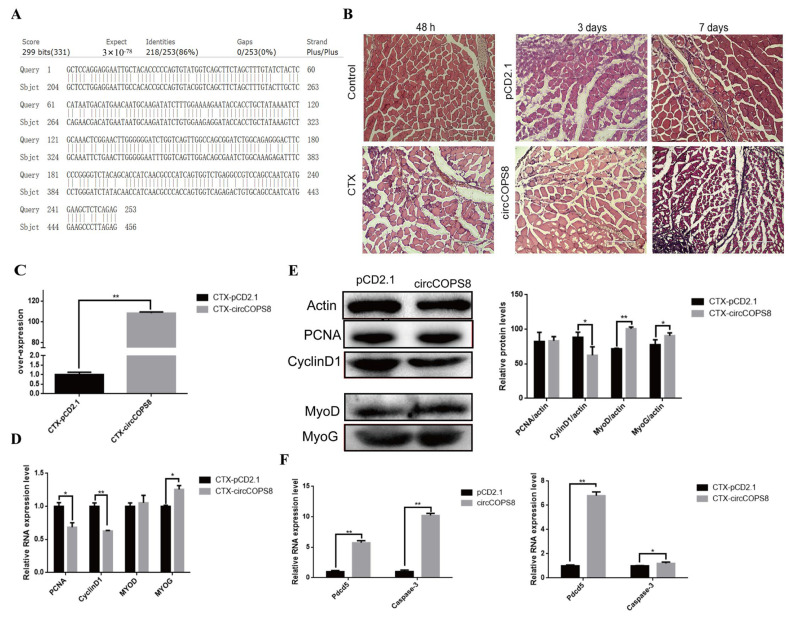
CirCOSP8 inhibits the repair of muscle injury in mice. (**A**) Comparison of mouse COPS8 genes and mRNA sequence comparison of circCOPS8. (**B**) Tibialis anterior tissue section of mouse muscle injury model (scale bar: 200 μm). (**C**) After in vivo injection of the plasmid pCD2.1-circCOPS8, the overexpression efficiency was measured (n = 3). (**D**) qPCR was used to detect the expression of proliferation and differentiation marker genes in buffalo myoblasts. (**E**) Western blotting and gray-scale statistical analysis of the marker genes. (**F**) qPCR of apoptosis marker genes. * *p* < 0.05 and ** *p* < 0.01.

Muscle damage repair and myogenic differentiation are closely related in mice. We used data from NCBI to compare the mRNA sequences of buffalo circCOPS8 and mouse COPS8, which showed 86% sequence identity (Figure 4A). In order to investigate the effect of circCOPS8 on muscle injury repair in mice, we constructed a mouse muscle injury model by injecting cardiotoxin (CTX) solution into the tibialis anterior muscle. When mouse muscle tissues were stained with H&E on the third and seventh days, the experimental group had more inflammatory cells than the control group, and the effectiveness of muscle fiber regeneration was poor (Figure 4B). Therefore, circCOPS8 may delay the repair process of damaged muscles in mice. RT-qPCR showed that circCOPS8 was overexpressed successfully in the tibialis anterior muscles in mice (Figure 4C). RT-qPCR and Western blotting showed that the expression levels of cyclinD1 and PCNA decreased, although the latter did not change significantly (Figure 4D,E). However, the expression levels of MyoD and MyoG increased markedly (Figure 4D,E). This suggested that circCOPS8 could enhance the expression of a differentiation marker gene while suppressing that of a proliferation marker gene in a mouse model of muscle injury. The findings above indicate that circCOPS8 significantly inhibits the proliferative capacity of buffalo myoblasts in vitro, and further delays the repair process while impeding cellular proliferation during muscle injury recovery in a mouse in vivo model. Subsequently, we investigated the impact of circCOPS8 on apoptosis. After overexpressing circCOPS8, we found that the expression of apoptotic marker genes such p53, Caspase-3 and Pdcd5 dramatically increased in buffalo myoblasts and muscle tissues of mice (Figure 4F). This indicated that circCOPS8 was able to induce apoptosis in buffalo and mouse myocytes.

### 3.5. GO and KEGG Analysis Highlights Critical Pathways in Muscle Development

**Figure 5:**
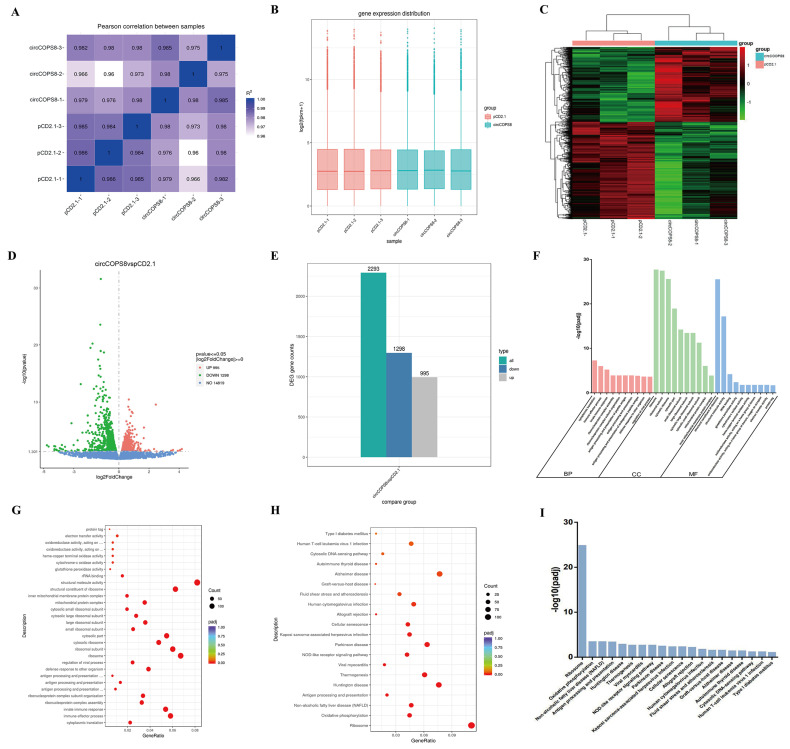
Transcriptome sequencing of circCOPS8 overexpressed in buffalo myoblasts. (**A**) A heatmap of correlation between samples. (**B**) Box diagram of sample gene expression distribution. (**C**) A cluster heat map of the differentially expressed gene. (**D**) A differential gene volcanic map. (**E**) Statistic histogram of the number of differential genes in the combination of difference comparison. (**F**) Histogram of GO function enrichment. (**G**) GO function enrichment bubble chart. (**H**) KEGG pathway enrichment bubble diagram. (**I**) KEGG pathway enrichment histogram.

To ensure the reliability of data analysis, the reads with sequencing linker containing N bases and low sequencing quality were filtered out (Supplementary File Figure S3A). Transcriptome sequencing data demonstrated satisfactory quality control metrics. All samples exhibited high Q30 scores (representing high-quality bases) (Supplementary File Figure S3B) and an adequate number of clean reads (Supplementary File Figure S3C), and alignment results against the reference genome showed that exon region coverage met expected standards (Supplementary File Figure S3D), confirming suitability for subsequent differential expression analysis. The samples exhibited a good correlation, and 2293 differential genes with annotation data were found, of which 1298 and 995 were respectively down- and up-regulated (Figure 5A–E). The differentially expressed genes (DEGs) were mapped to 113 GO terms, mainly including ribonucleoprotein complex assembly (GO: 0022618), immune response process (GO: 0002252) and antigen processing and presentation (GO: 0019882) (Figure 5F,G). We performed KEGG analysis to reveal the biological functions of these DEGs. The results showed that the DEGs were mainly enriched in the ribosomal (bbub03010) and oxidative phosphorylation (bbub00190) pathways (Figure 5H,I). Among them, the pathways related to cell proliferation and differentiation included cell senescence (bbub04218), cell cycle (bbub04110), DNA replication (bbub03030), longevity regulation (bbub04211) and apoptosis (bbub04215). This closely aligns with our observed phenotype of circCOPS8 inhibiting cell proliferation, suggesting that circCOPS8 may exert its function by modulating these core pathways.

### 3.6. CircCOPS8 Inhibits the Proliferation of Myoblasts by Binding to IGF2BP3 and Promoting Tumor Suppressor Gene Expression

**Figure 6:**
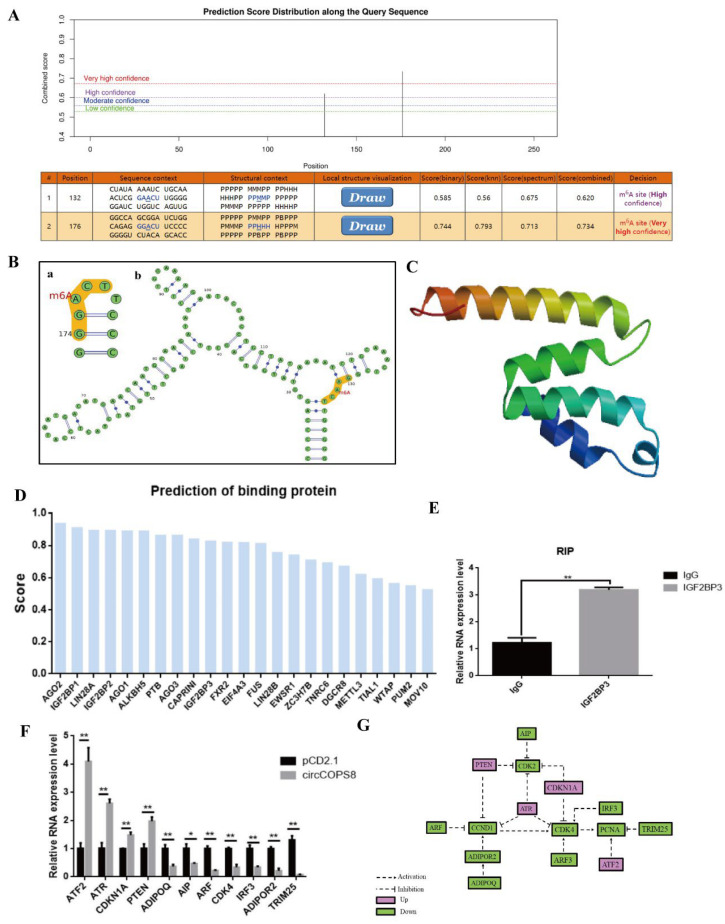
Mechanism of circCOPS8. (**A**,**B**) Prediction of the m6A methylation sites ((**a**): m^6^A modification site. (**b**): RNA secondary structure). (**C**) The protein tertiary structure. (**D**) Binding protein prediction. (**E**) RIP-qPCR of IGF2BP3. (**F**) qPCR verification of the differential genes. (**G**) The gene pathway map. * *p* < 0.05, ** *p* < 0.01.

CircCOPS8 may function as a molecular sponge, a protein scaffold, and a coded short peptide since RNA-FISH studies indicate that it is localized in the cytoplasm. We predicted the potential mechanism of circCOPS8 via the online website, as follows. Firstly, the results showed that circCOPS8 had almost no bound miRNAs, while it may contain an ORF region [25]. The confidence in the finding was not high, implying that circCOPS8 was likely to lack IRES locus (Supplementary File, Table S10) [26]. We further predicted the circCOPS8 m6A modification site of circCOPS8 (Supplementary File, Table S9), and it suggested that circCOPS8 contained two potential m6A binding sites (Figure 6A,B) [27]. The amino acid sequence of circCOPS8 exhibited high similarity to that of 6R6H-HY and other proteins, all of which were components of the COP9 signal complex (Figure 6C) [28].

In conclusion, circCOPS8 may encode a small peptide through its m6A modification site, and its function is probably similar to that of protein encoded by its source gene, COP9. In addition, circCOPS8 may bind to proteins such as AGO2 and IGF2BP3 (Figure 6D) [26,29]. Since IGF2BP3 is related to cell proliferation and m6A, we explored the possibility that it may bind to circCOPS8. RIP-qPCR results showed that circCOPS8 was capable of binding with IGF2BP3 (Figure 6E). The upstream pathway genes of proliferation marker genes were identified through the KEGG website and subsequently compared with the differential genes of the transcriptome [30], and a gene action pathway map was constructed. RT-qPCR was then used to verify the genes in the mechanism map. The differential genes consistent with the transcriptome sequencing results were then drawn into a gene pathway map (Figure 6F,G). Based on the results, we hypothesize that circCOPS8 may suppress the expression of cell proliferation-related genes by activating certain tumor suppressor genes. A possible scenario could be that ATR simultaneously inhibits the expression of CCND1 and CDK2/4, and PTEN inhibits the expression of PCNA and CCND1. This needs to be verified by future experiments.

## 4. Discussion

With the development of high-throughput sequencing technology, an increasing number of studies on non-coding RNAs have been reported. CircRNAs are a type of non-coding RNAs characterized by a ring structure, which increases their bio-stability and endows them with longer half-lives in biological systems. Buffalo is a specialty species of Guangxi, but the meat yield of buffalo is lower than that of yellow cattle and other species. In this study, we used RNA-seq technology to screen circRNAs in the longissimus dorsi muscles of buffaloes and compared them with existing RNA-seq data from cattle, aiming to identify differentially expressed molecules associated with muscle tissue differentiation and proliferation. CircCOPS8 was subsequently selected for further investigation.

Verification of the authenticity of circRNAs is an important step in circRNA studies, and we have successfully verified that circCOPS8 is an authentic molecule by using the RNase digestion assay. The COP9 signalsome subunit 8 (COPS8) is one of the eight subunits of the COP9 signalsome (CSN), and it is a key multifunctional evolutionarily conserved protein complex in cells that is essential to the wellbeing of all plants and animals. We hypothesized that circCOPS8, derived from COPS8, may play an important role in the cell cycle.

Subsequently, we transfected plasmids pCD2.1 and pCD2.1-circCOPS8 into buffalo myoblasts, and the results of EdU, CCK-8 and Western blotting assays showed that circCOPS8 promoted the differentiation and inhibited the proliferation of buffalo myoblasts. Using a muscle injury mouse model, we further confirmed that circCOPS8 exerted similar functions in mouse muscle cells; i.e., circCOPS8 promoted the differentiation of mouse myoblasts and inhibited their proliferation. H&E staining of mouse tibialis anterior muscle tissues showed that circCOPS8 inhibited the repair of muscle injury in mice. Additional experiments also showed that circCOPS8 promoted apoptosis in both buffalo and mouse myoblasts. We then explored the effect of circCOPS8 on apoptosis, and the results showed that it significantly promoted the expression of apoptotic marker genes.

The function of a circRNA varies depending on its subcellular localization. CircRNAs mainly function in the nucleus by regulating gene transcription. By adsorbing proteins, miRNAs and small peptides that code for genes, circRNAs may also operate in the cytoplasm. As it is found in the cytoplasm, circCOPS8 it is predicted to have a potential mode of operation through a number of sources. The findings suggested that circCOPS8 may encode a small peptide, potentially through adsorbing to proteins or binding to m6A sites; however, this remains a bioinformatic prediction requiring experimental validation via techniques such as ribosome profiling (Ribo-seq) and mass spectrometry. CircCOPS8 binding proteins can be divided into three categories: DGCR8, AGO, and other proteins involved in the synthesis and splicing of miRNAs [31,32]. Proteins such as FIF4A3 and TIAIL1 are linked to the apoptosis of cancer cells, while proteins such as FUS are involved in gene repair [33,34]. IGF2BP3, a protein implicated in cell proliferation and muscle development, was shown by RIP-qPCR results to bind to circCOPS8, thereby inhibiting cell growth. In addition, the small peptide encoded by circCOPS8 may be involved in the formation of the COPS9 signal complex.

Using transcriptome data, we identified a route whereby circCOPS8 suppresses cell growth, and this was confirmed by RT-qPCR. Based on the research results, we hypothesize that circCOPS8 inhibits the expression of cell proliferation markers by activating tumor suppressor genes. For instance, PTEN suppressed CDK4 and CCND1 expression while ATR inhibited the expression of CDK2/4 and CCND1. ATR is a key gene that arrests the cell cycle, while PTEN regulates the synthesis of a specific protein [35,36,37,38,39], which acts as a tumor suppressor to regulate cell cycle division by preventing abnormal cell growth and uncontrolled division.

Buffaloes have better disease resistance than cattle, and the latter are known to lack circCOPS8 expression in their longissimus dorsi muscles. Our results demonstrated that co-expression of circCOPS8 and IGF2BP3 inhibited cell proliferation. By promoting the expression of tumor suppressor genes, circCOPS8 prevented the production of genes relevant to the cell cycle. Thus, it is plausible that circCOPS8 contributes to the superior disease resistance of buffaloes, a hypothesis that requires further investigation. Overexpression of circCOPS8 inhibits the proliferation of buffalo myoblasts while promoting their differentiation, which may potentially affect muscle fiber density, size, or composition by limiting the myoblast pool and its proliferative capacity, thereby contributing to the suboptimal texture of buffalo meat. Future studies are needed to further validate this hypothesis by investigating the correlation between circCOPS8 expression levels and specific meat quality parameters (e.g., tenderness, shear force) in buffaloes. In addition, the detailed mechanism for circCOPS8 production and its role also needs to be addressed in future studies. In conclusion, we explored some of the functions and mechanisms of circCOPS8, and this study will provide a theoretical basis for understanding the role of circRNAs in regulating buffalo muscle development.

## 5. Conclusions

CircCOPS8 promoted the differentiation and apoptosis of buffalo myoblasts while inhibiting their proliferation. Its inhibitory effect on cell proliferation is mediated by binding to IGF2BP3 and promoting ATR gene expression.

## Data Availability

The RNA-seq data of the longissimus dorsi muscle of buffalo and cattle were sent to the NCBI database, and the data number is SAMN21421329. The online version contains Supplementary Materials available at https://orcid.org/my-orcid?orcid=0000-0002-5604-0592, accessed on 19 March 2026. All data are available upon request from the corresponding authors.

## Supplementary Materials

The following supporting information can be downloaded at https://www.mdpi.com/article/10.3390/ani16071017/s1: Figure S1. CircCOPS8 sequence in the buffalo genome. Figure S2. RNA-FISH probe primer. Figure S3. Quality control of transcriptome sequencing. Table S1. Primers. Table S2. Primers for vector construction. Table S3. Transcriptome sequencing quality control data. Table S4. Up-regulated and down-regulated genes. Table S5. GO enrichment analysis. Table S6. KEGG enrichment analysis. Table S7. Binding protein prediction—CatRAPID. Table S8. Binding protein prediction—RBPsuite. Table S9. Prediction of m6A binding sites. Table S10. Prediction of IRES locus.

## Abbreviations

The following abbreviations are used in this manuscript:

CircRNA	Circular RNA
MiRNA	MicroRNA
RNA-seq	RNA Sequencing
qPCR	Quantitative Polymerase Chain Reaction
MRFs	Myogenic Factors 5
Myf6	Myogenic Factors 6
MyoD	Myoblast Determination Protein
MyoG	Myogenin
Pax3	Paired Box 3
Pax7	Paired Box 7
SiRNA	Short Interfering RNA
CeRNA	Competing Endogenous RNA
RNA-FISH	RNA-fluorescence in Situ Hybridization
BSA	Albumin from Bovine Serum
WB	Western Blot
IRES	Internal Ribosome Entry Site
IGFs	Insulin-like Growth Factor
RBP	RNA Binding Protein
PCNA	Proliferating Cell Nuclear Antigen
CDK2	Cyclin Dependent Kinase 2
ATM	Ataxia Telangiectasia-mutated
Map3k20	Protein Kinase Kinase Kinase 20
GO	Gene Ontology
KEGG	Kyoto Encyclopedia of Genes and Genomes
