# Supplementary figures and images for: CircCOPS8 Inhibits the Proliferation of Buffalo Myoblasts by Binding to IGF2BP3 and Promoting ATR Gene Expression
Source: Animals (Basel). 2026 Mar 26;16(7):1017. doi: 10.3390/ani16071017 (PMC13071993; doi:10.3390/ani16071017)

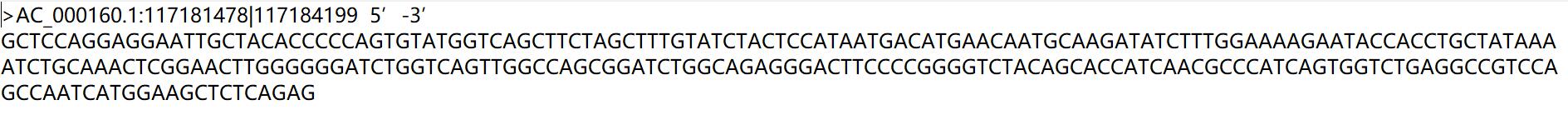

Supplement: Supplementary file 1 [file animals-16-01017-s001.zip › Figure S1 CircCOPS8 sequence in the bovine genome.jpg]

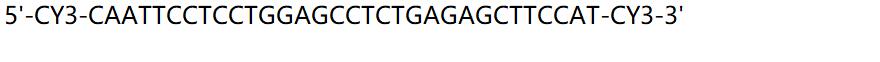

Supplement: Supplementary file 1 [file animals-16-01017-s001.zip › Figure S2 RNA-FISH probe primer.jpg]

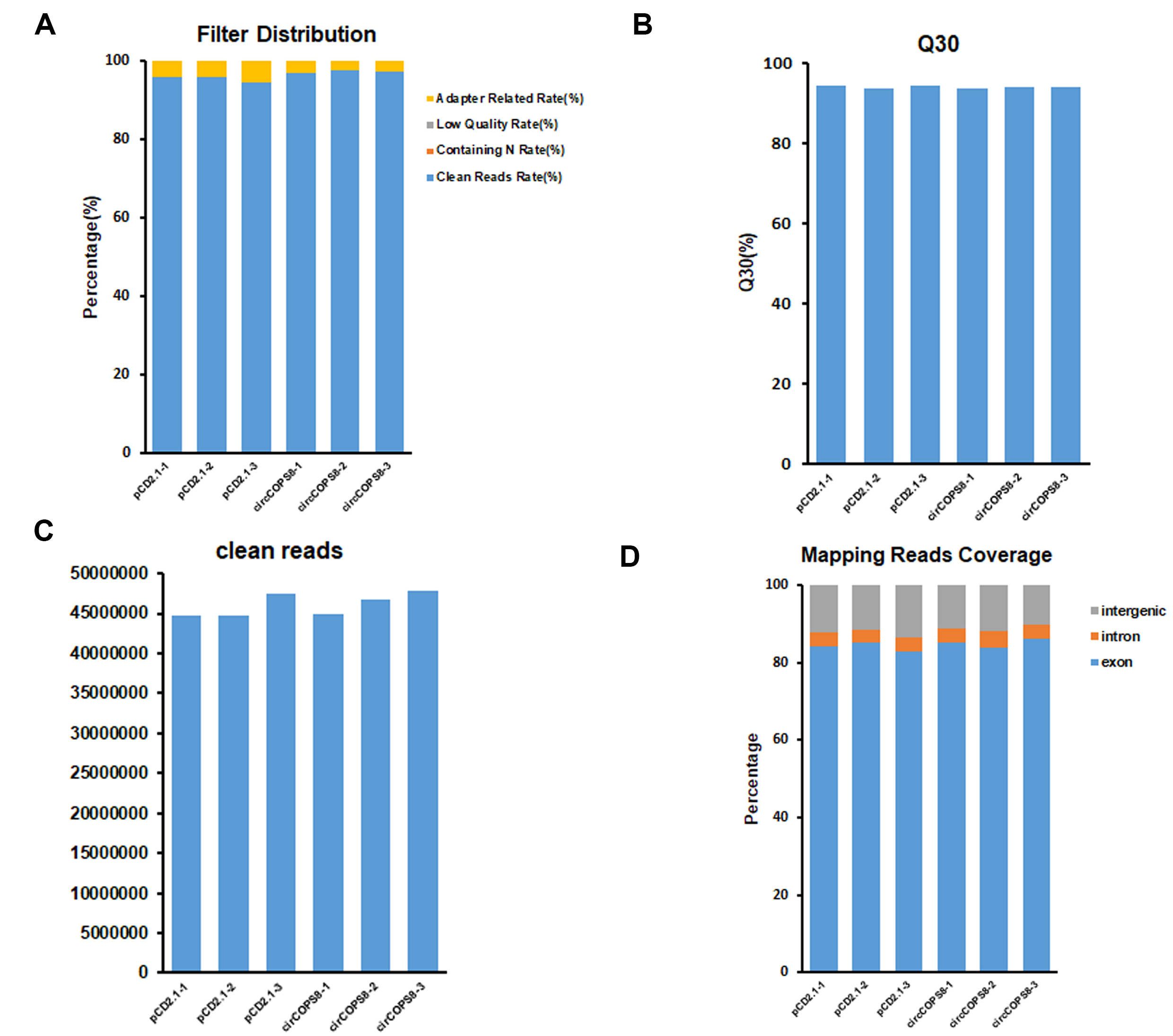

Supplement: Supplementary file 1 [file animals-16-01017-s001.zip › Figure S3 Quality control of transcriptome sequencing.jpg]
